# Intercropping System and N_2_ Fixing Bacteria Can Increase Land Use Efficiency and Improve the Essential Oil Quantity and Quality of Sweet Basil (*Ocimum basilicum* L.)

**DOI:** 10.3389/fpls.2020.610026

**Published:** 2020-12-23

**Authors:** Sajad Kordi, Saeid Zehtab Salmasi, Jalil Shafagh Kolvanagh, Weria Weisany, Dennis A. Shannon

**Affiliations:** ^1^Department of Ecophysiology, Faculty of Agriculture, University of Tabriz, Tabriz, Iran; ^2^Department of Agriculture and Food Science, Science and Research Branch, Islamic Azad University, Tehran, Iran; ^3^Department of Crop, Soil and Environmental Sciences, Auburn University, Auburn, AL, United States

**Keywords:** basil, cropping system, essential oil composition, fertilizer, integrated management

## Abstract

Intercropping fodder plants with medicinal plants, in addition to enhancing productivity, can remarkably reduce the population of weeds, pests and diseases and for naturally meeting of livestock medicinal needs. Two experiments were conducted to evaluate biological yield, essential oil (EO) composition and yield of sweet basil (*Ocimum basilicum* L.) treated with N_2_ fixing bacteria in additive intercropping with forage maize during the 2018 and 2019. Treatments were arranged in factorial split-plot-in time in randomized complete block design with three replications. The factors were 100% chemical fertilizer (N), N_2_ fixing bacteria (*Azospirillum brasilense* and *Azotobacter chroococcum*), integration of N_2_ fixing bacteria + 50% nitrogen chemical fertilizer and control. The cropping pattern factor included of sole cropping basil and the additive intercropping of maize + 25% basil, maize + 50% basil, maize + 75% basil, and maize + 100% basil. The results indicated that the highest essential oil yield (30.8 kg ha^−1^) and essential oil percentage (0.75%) were obtained in sole cropping with *A. brasilense* and *A. chroococcum* + 50% chemical nitrogen fertilizer application in second harvest in 2019. In both cropping systems, the N_2_ fixing bacteria application significantly increased fresh and dry yield and land equivalent ratio (LER) as compared to control plants. In both years of experiments could remarkably vary depending on type of treatment. In both years, eight constituents including methyl chavicol (17.24–51.28%), Z-citral (neral) (8.33–24.3%), geranial (10.2–31.3%), (E)-caryophyllene (1.05–5.64%), α-*trans*-bergamotene (0.53–1.7%), α-humulene (0.4–1.69%), germacrene-D (0.2–1.88%), and (Z)-α- bisabolene (1.16–3.86%) were the main constituents of EO. The highest content of methyl chavicol was found through sole cropping of sweet basil with nitrogen chemical fertilizer followed by sole cropping of sweet basil with an integration of *A. brasilense* and *A. chroococcum* + 50% nitrogen chemical fertilizer in 2018 and 2019. Intercropping system and N_2_ fixing bacteria can be effective in reducing chemical fertilizer consumption and environmental pollution and achieving the sustainable agriculture goals.

## Introduction

Since the green revolution and the introduction of fertilizer-consuming cultivars, the consumption of chemical fertilizers has been widely increased. Soil fertility improvement is mainly achieved through the use of chemical fertilizers, which their high consumption is a threat to environment and human health (Kordi, 2017). In this regard, some issues concerning soil like erosion, salinity, acidification, and decline in qualitative and quantitative properties of surface-area soils as well as other issues such as contaminating surface and groundwater, devastating biodiversity, reduced soil biological activities, and finally increasing cost of crop production are consequences of illogical using of chemical fertilizers (Kordi et al., 2017). Some conditions, such as climatic and nutrient factors suitable for plant growth, are the most important factors affecting growth of medicinal plants and their active components quantitatively and qualitatively (Street, 2012). Therefore, alternative sources of fertilizer and the use of organic fertilizers (e.g., N_2_ fixing bacteria) are considered as sustainable agriculture options to improve soil quality in modern agriculture (Chen et al., 2014; Meena et al., 2015).

The utilization of bio-fertilizers (e.g., free-living N-fixing bacteria) is of particular importance in the agriculture sector due to their potential role in healthy food, improving crop yield, and decreasing greenhouse emissions (Ghilavizadeh et al., 2013; Raei et al., 2015). Raei et al. (2015) showed that the seeds of sweet basil inoculated with *Azospirillum* produced plants with higher fresh and dry weights, height, and more lateral branches as compared to controls. Ghilavizadeh et al. (2013) showed that inoculating the seeds of fennel with *Azotobacter* and *Azospirillum* could increase seed essential oil (EO) content.

In industrial agriculture, one of the major ways to increment livestock production is to utilization a diet having some cereal grains such as maize and barley, or silage plants and many types of concentrates containing dietary supplements and chemical content designed to stimulate animal growth and prevent diseases. The improper application of these compounds is associated with outbreaks of many metabolic diseases such as liver abscesses and acidosis in ruminants, and finally causes many problems (Kordi et al., 2017). Nowadays, these problems have led to organic crops production is considered as an option to decrement the negative effects of chemical fertilizers (Aguilera et al., 2013; Aires et al., 2013). In some countries where organic agriculture is practiced, emphasis has been placed on availability of some medicinal plants such as chicory (*Cichorium intybus* L.) and caraway (*Carum carvi* L.) in pasture that is grazed by livestock for naturally meeting their medicinal needs (Kordi et al., 2017). In this regard, “medicinal forage” idea was introduced. From the standpoint of forage value, although some medicinal plants fed solitarily to livestock have resulted in forage unpalatability or with high amounts of anti-nutritional compounds (such as coumarin, phenolic compounds, and prussic acid), intercropping with ordinary forage plants gives them high value as medicinal forage through efficient, scientific management (Kordi et al., 2017). Intercropping fodder plants with medicinal plants, in addition to enhancing productivity, can remarkably reduce the population of weeds, pests, and diseases and increase plant tolerance to biotic and abiotic stresses. By intercropping sweet basil with maize, Kordi (2017) obtained highest land equivalent ratio (LER) (1.566) with a combination of maize + 100% stands of basil and N_2_ fixing bacteria.

Sweet basil (*Ocimum basilicum* L.) is an annual herbaceous plant in the Lamiaceae family, native to Asia, Africa, America, and the subtropics (Roman, 2012; Borloveanu, 2014). Many aromatic plants including basil are rich in secondary metabolites (Srivastava et al., 2014; Li et al., 2017). Having high commercial values, these secondary metabolites are exploited largely as flavors, fragrances and pharmaceuticals (Al-Maskri et al., 2011).

Many studies documented that EO of sweet basil have antifungal, insecticidal (Hossain et al., 2014a,b), antibacterial, and antioxidant (Karagözl et al., 2011) properties. The chemical composition of sweet basil EO depends on genetic, season, environmental factors, and the plant growth stage (Bilal et al., 2012). Padalia et al. (2014) has reported linalool, methyl chavicol, methyl eugenol, eugenol, and geraniol as dominant components in the basil EO. Since there is limited information about the suitability of medicinal plants especially sweet basil as intercrops in maize in Iran, therefore this study was conducted to evaluate biological yield, yield and chemical composition of sweet basil EO treated with nitrogen fertilizers (N_2_ fixing bacteria, chemical and integrated) in additive intercropping with forage maize.

## Materials and Methods

### Location and Plant Materials

This research was carried out in the Experimental Farm of Faculty of Agriculture, Lorestan University, Iran (33°29′N, 48°22′E and altitude 1,125 m), during 2017–2018 and 2018–2019 growing seasons. Weather conditions during the experimental period are shown in Table 1. Physicochemical properties of soil at the depth 0–40 cm are presented in Table 2. Different cropping patterns included: sole cropping pattern of sweet basil (80 plants per m^2^), and the additive intercropping of sweet basil at 25% (20 sweet basil plants m^−2^), 50% (40 sweet basil plants m^−2^), 75% (60 sweet basil plants m^−2^), and 100% basil (80 sweet basil plants m^−2^) stand into maize at 10 plants m^−2^. Crops seed were sown in plots whose area was 5 m^2^, consisting of five 2-m rows spaced 50 cm apart. Both crops were planted on the same day in early May 2018 and 2019. The distance between plants on the row was 20 cm for maize (*Zea mays* L. cv. S.C. 704) and was 2.5, 3.3, 5, and 10 cm for different ratio of sweet basil, respectively. All plots were irrigated immediately after sowing and subsequent irrigations were performed every 5 days. Hand weeding was done as needed.

**Table 1:** Khorramabad meteorological station monthly statistics in the experiment period in 2018 and 2019.

**Month**	**Precipitation (mm)**		**Number of rainy days**		**Maximum temperature (**^****°****^**C)**		**Minimum temperature (**^****°****^**C)**		**Average temperature (**^****°****^**C)**	
	**2018**	**2019**	**2018**	**2019**	**2018**	**2019**	**2018**	**2019**	**2018**	**2019**
Apr	51.4	86.9	8	9	20.9	21.2	5.2	6.1	13.2	13.7
May	14.5	22.8	5	8	28.2	29.4	10.5	10.7	19.4	20
Jun	0.2	2	1	2	34.8	37.3	14.6	15.6	24.7	26.4
Jul	0	0.2	0	1	40	40.2	19.3	20.4	29.6	30.2
Aug	0	0	0	0	40.5	41	19.4	20.4	30	30.7
Sep	0	17	0	1	36.9	36.2	15	17.4	25.6	26.8

**Table 2:** Some physical and chemical properties of the soil of experimental area.

**Year**	**Soil texture**	**Clay (%)**	**Silt (%)**	**Sand (%)**	**pH**	**EC (dS m^**−1**^)**	**Total N (%)**	**Available P (ppm)**	**Available K (ppm)**
2018	Clay loam	32.16	42	25.84	7.17	0.459	0.302	8	390
2019	Clay loam	31.52	41.5	26.98	7.36	0.536	0.285	6	356

### Experimental Design

The main experimental design of this study was a factorial experiment based on Randomized Complete Blocks Design with three replications. However, due to three harvest times in each season for basil plants, the basil traits except for fresh and dry yields (which were total yield of three harvests) were statistically analyzed based on factorial split-plot-in time experiment. Fertilization factor consisted of F_1_: control (without nitrogen fertilization); F_2_: 100% chemical fertilizer (N); F_3_: nitrogen-fixing bacteria (*Azospirillum brasilense* and *Azotobacter chroococcum*) and F_4_: integration of nitrogen-fixing bacteria + 50% nitrogen chemical fertilizer. Cropping pattern factor included sole cropping of basil (I_1_) and the additive intercropping of maize + 25% basil (I_2_), maize + 50% basil (I_3_), maize + 75% basil (I_4_), and maize + 100% basil (I_5_) (Kordi et al., 2017). Basil was harvested three times each season. The experiment was repeated at a different site in the second season.

### Fertilizer and Microbial Inocula

According to soil test, 150 kg triple superphosphate ha^−1^ and 50 kg potassium sulfate ha^−1^ were applied before cultivation. In application of 100% chemical fertilizer (F_2_), 375 kg N ha^−1^ was used as urea. The half of the nitrogen amount was added to F_2_ and F_4_ treatments (187.5 and 93.7 kg N ha^−1^, respectively) with the last plow before sowing. The rest of the nitrogen was used in two stages: the eight-leaf stage of maize and before the start of tassel formation in the plots. The N_2_ fixing bacteria were *Azospirillum brasilense* and *Azotobacter chroococcum* strains. Both strains of bacteria consisted of 10^8^ CFU ml^−1^ inoculant which were provided from Mehr Asia Biotechnology Company, Tehran, Iran. Following cultivation, the seeds were completely soaked with the N_2_ fixing bacteria and kept in the shade for half an hour in to dry and be ready for planting. The liquid N_2_ fixing bacteria (*A. brasilense* and *A. chroococcum*) applied at 2 L ha^−1^ (Kordi et al., 2017).

### Traits Measurement

The traits measured in this research included fresh and dry yields, EO percentage, chemical composition and yield of sweet basil EO.

#### Fresh and Dry Yields

The sweet basil was harvested three times each season in early flowering stage in July, August, and September. Samples of 1 m length were taken from the center of two rows located in middle of each plot. Plants were cut above ground and transferred into a lab to measure fresh weight. To measure dry weight, the samples were dried in an oven at 75°C for 72 h and then weighed. For determination of EO content, the aerial parts of sweet basil plants were dried naturally in the shade.

#### Essential Oil Isolation

Fifty grams of naturally dried aerial parts were sampled for analysis. Woody parts of plants were separated and then remainder parts hydro-distilled for 3.5 h by a Clevenger-type apparatus (Weisany et al., 2016a,b). The obtained EO was dehydrated using anhydrous sodium sulfate and stored in sealed vials at 4°C, until further analyses. To measure main constituents of EO, all treatments belonged to a given repetition were chosen in second harvest (Weisany et al., 2015).

##### Gas Chromatography-Mass Spectrometry (GC-MS)

The EO samples were analyzed in a gas chromatograph Agilent model 7890 using HP-5MS column (30 m × 0.25 mm, 0.25 μm in thickness). The oven temperature was programmed from 50°C (held for 2 min) and increased to 240°C at a rate of 3°C/min then 240–300°C at a rate of 15°C/min. The constant flow rate of helium as carrier gas was 1 mL/min. The mass spectra were recorded on electron ionization (EI) mode, with ionization energy of 70 eV. The temperature at the injection site was 290°C. The identification of chemical constituents was done based on the retention indices (calculated using from C8 to C20 alkanes) and through analyzing the mass spectra compared to a computer databank (Wiley 7 and Nist 62) (Adams, 2007; Carneiro et al., 2017).

#### Land Equivalent Ratio (LER)

Land equivalent ratio is an index for comparing and estimating the advantage of different methods of intercropping compared to monocropping. To calculate the LER, the intercrop yield of one culture is divided by the yield of the pure stand (Mead and Willey, 1980). In present research, seed weight is considered as yield parameter.

$$\begin{array}{l} \text{LER}=\left(\text{Yab}/\text{Ya}\right)+\left(\text{Yba}/\text{Yb}\right) \end{array}$$

where Ya and Yb are the sweet basil and maize yields in sole cropping and Yab and Yba are the yields of sweet basil and maize in intercropping, respectively. LER values >1 shows the superiority of intercropping system in environmental resources consumption for growth and production of plant compared to sole cropping, and when LER <1 resources are used more efficiently in monocropping than in intercropping system (Vandermeer, 1989).

### Data Analysis

SAS and MSTATC softwares were applied to analysis of variance (ANOVA) and comparison of means, respectively. The mean data were compared using Duncan's multiple range test at *p* ≤ 0.05. The graphs were drawn by Excel and error bars were assigned on the basis of standard deviation (SD).

## Results and Discussion

### Fresh and Dry Yields of Basil

The result showed that the highest fresh and dry yields of sweet basil were obtained by sole cropping of sweet basil and using integration of *A. brasilense* and *A. chroococcum* + 50% nitrogen chemical fertilizer in second year of experiment (Table 3). Both years did not have the same effect on the mentioned traits, due to difference between climatic conditions in those periods, such that appropriate climate in early of second year caused plants to have a better establishment and growth consequently, led to more photosynthesis activities and yield. The higher fresh and dry yields in sole cropping of sweet basil can be attributed to the homogeneous environment in monoculture system (Amani-Machiani et al., 2018). This demonstrated that the interspecific competition in the sweet basil + forage maize intercropping system was higher than the intraspecific competition in sole cropping system (Xie and Kristensen, 2017; Amani-Machiani et al., 2018). On the other hand, the high competitive ability of forage maize compared to sweet basil in different intercropping patterns led to a significant decrement in sweet basil yield, likely due to shade from the maize. In a study, it was found that among different cropping patterns, higher fresh and dry yields of sweet basil were gained by its sole cropping relative to its intercropping with maize (Bagheri et al., 2014). Amani-Machiani et al. (2018) indicated that the peppermint biomass in intercropping with faba bean (*Vicia faba* L.) was significantly less than sole crop of peppermint. As nitrogen enhances plant's vegetative growth and consequently improves its yield, accordingly the more nitrogen is used by plants, by impacting on physiological processes, increases photosynthesis activities and produces more assimilate, biomass, and eventually yield. Due to effects of *A. brasilense* and *A. chroococcum* on nitrogen fixing and secreting growth activator in comparison to control, the yield of sweet basil was significantly increased by application *A. brasilense* and *A. chroococcum* (Ghilavizadeh et al., 2013; Pešakovic et al., 2013).

**Table 3:** Fresh and dry yield of maize and basil as affected by sole and intercropping systems in 2018 and 2019.

**Cropping systems**		**Nitrogen sources**	**Fresh yield**				**Dry yield**			
			**2018**		**2019**		**2018**		**2019**	
			**Maize (Ton/ha)**	**Basil (kg/ha)**	**Maize (Ton/ha)**	**Basil (kg/ha)**	**Maize (Ton/ha)**	**Basil (kg/ha)**	**Maize (Ton/ha)**	**Basil (kg/ha)**
	M10	F1	59 ± 2.63	–	64.1 ± 0.55	–	16.5 ± 0.73	–	17.9 ± 0.14	–
	M10	F2	74.8 ± 2.3	–	76.6 ± 0.91	–	20.6 ± 0.49	–	21.3 ± 0.52	–
	M10	F3	63.1 ± 1.93	–	70.7 ± 3.74	–	17.6 ± 0.26	–	19.7 ± 0.77	–
	M10	F4	68.8 ± 2.31	–	73.7 ± 1.01	–	19.4 ± 0.16	–	20.7 ± 0.12	–
Sole cropping	B80	F1	–	24,326 ± 399	–	22,920 ± 498	–	4,236 ± 59	–	3,995 ± 774
	B80	F2	–	36,529 ± 1,503	–	46,497 ± 1,206	–	6,327 ± 256	–	8,036 ± 208
	B80	F3	–	29,514 ± 1,239	–	30,867 ± 2,478	–	5,125 ± 214	–	5,356 ± 425
	B80	F4	–	37,259 ± 313	–	47,591 ± 1,403	–	6,451 ± 54	–	8,224 ± 240
Intercropping	M10/B20	F1	56.3 ± 4.36	7,769 ± 230	62.5 ± 3.54	7,183 ± 91	16 ± 0.94	1,335 ± 41	17.5 ± 1.47	1,231 ± 16
	M10/B20	F2	72.1 ± 1.82	8,909 ± 155	76.6 ± 2.76	8,649 ± 307	20.3 ± 0.4	1,530 ± 29	21.1 ± 0.39	1,484 ± 53
	M10/B20	F3	63.2 ± 2.54	8,986 ± 171	68.2 ± 2.66	8,295 ± 231	17.9 ± 0.93	1,541 ± 28	19.1 ± 0.84	1,424 ± 37
	M10/B20	F4	68 ± 4.3	9,178 ± 378	72.1 ± 1.47	8,913 ± 497	19.6 ± 0.39	1,574 ± 65	20.3 ± 0.57	1,529 ± 85
	M10/B40	F1	52.3 ± 2.52	11,307 ± 652	57.5 ± 2.92	10,371 ± 64	15.2 ± 0.44	1,938 ± 112	16.5 ± 0.43	1,778 ± 11
	M10/B40	F2	66.7 ± 1.01	14,977 ± 577	74.1 ± 1.98	15,649 ± 355	19.4 ± 0.04	2,568 ± 97	21.3 ± 0.33	2,683 ± 61
	M10/B40	F3	61.4 ± 4.05	13,722 ± 500	65.6 ± 1.85	14,235 ± 472	17.8 ± 0.5	2,352 ± 86	18.8 ± 0.57	2,440 ± 81
	M10/B40	F4	64.8 ± 3.3	15,161 ± 175	71.6 ± 1.53	16,191 ± 222	19 ± 0.56	2,600 ± 31	20.6 ± 0.33	2,777 ± 37
	M10/B60	F1	50 ± 3.10	13,688 ± 175	56.1 ± 2.84	13,761 ± 345	14.9 ± 0.19	2,347 ± 29	16.4 ± 0.86	2,360 ± 60
	M10/B60	F2	63.4 ± 4.1	20,311 ± 324	69.6 ± 1.39	22,702 ± 837	18.6 ± 1.14	3,484 ± 57	20.3 ± 2.48	3,892 ± 143
	M10/B60	F3	57 ± 3.8	17,779 ± 209	61.7 ± 4.5	19,631 ± 396	16.9 ± 0.55	3,048 ± 36	18 ± 1.24	3,365 ± 68
	M10/B60	F4	61.4 ± 0.89	20,960 ± 740	69.8 ± 2.07	22,829 ± 557	18.2 ± 0.27	3,593 ± 127	20.4 ± 0.27	3,915 ± 96
	M10/B80	F1	47.9 ± 0.38	16,524 ± 642	49.1 ± 1.14	15,720 ± 180	14.4 ± 0.37	2,833 ± 110	14.8 ± 0.27	2,697 ± 31
	M10/B80	F2	61.1 ± 0.67	23,248 ± 321	67.8 ± 3.32	25,476 ± 1,678	18.1 ± 0.24	3,986 ± 54	19.9 ± 1.1	4,367 ± 288
	M10/B80	F3	53.1 ± 3.82	20,044 ± 451	58.1 ± 1.89	21,260 ± 317	15.8 ± 0.97	3,438 ± 78	17.3 ± 0.42	3,646 ± 56
	M10/B80	F4	58.6 ± 1.82	23,407 ± 255	66.2 ± 3.45	25,888 ± 519	17.4 ± 0.22	4,013 ± 44	19.6 ± 0.89	4,438 ± 89

*F1, control; F2, 100% chemical fertilizer (N); F3, Azospirillum brasilense and Azotobacter chroococcum; F4, integration of A. brasilense and A. chroococcum + 50% nitrogen chemical fertilizer*.

### Fresh and Dry Yields of Maize

The highest fresh and dry yields of maize were achieved in second year (Table 3). The effect of two growing seasons on these traits was not the same and the difference between the growing seasons was significant. Better climatic conditions in the beginning of the growing season provided suitable seedling establishment, photosynthesis and yield for maize plants in the second year.

The data presented in Table 3 showed that among the different intercropping systems the highest fresh and dry yields of maize were obtained from sole cropping pattern. Among the different intercropping systems, sole cropping pattern in terms of dry yield of maize had no significant difference with maize + 25% basil treatment. Due to high plant density of basil intercropped with maize and competition for water and nutrients, the fresh and dry yields of maize in maize + 100% basil pattern was apparently decreased as compared to maize's monoculture. Best utilization of nutrients, moisture, space, and solar energy can be derived through mono cropping system (Aiyer, 1963). Bagheri et al. (2014) reported that fresh and dry weight of leaves, stem, and ear in sole culture of maize were more than intercrops. Increasing the proportions of sweet basil and/or borage decreased the weight of leaves, stem, and ear of maize. Higher yield of sole culture compared to intercrop may be due to minimal disruption of the plants habitat (Banik et al., 2006).

The highest and lowest fresh and dry yields of maize have shown by nitrogen chemical fertilizer and control (without-fertilizer) treatments, respectively (Table 3). Application of *A. brasilense* and *A. chroococcum* resulted in 12% extra dry yield of maize, compared with the control. It seems that application nitrogen fertilizer plays a significant role in increasing plant's vegetative growth and consequently paves the way for increasing the yield of forage corn. An increase in the rate of applied nitrogen and also its effect on physiological processes resulted in more photosynthesis, assimilation, dry matter and yield. In other words, due to nitrogen-fixing and the effects of nitrogen-fixing bacteria on secreting growth regulator as well as stimulating plant's growth, the forage corn's yield was increased through application of nitroxin biofertilizer as compared to control. The positive role of nitrogen on qualitative and quantitative traits of corn such as dry weight has been proved (Cox et al., 1993). In this respect, Nanda et al. (1995) also showed that inoculating corn's seeds with nitrogen-fixing bacteria under field conditions caused to increase the yield compared with different levels of nitrogen fertilizer. By 2-year planting two types of corn seeds in the field (i.e., the inoculated seeds with free living N-fixing bacteria and non-inoculated seeds as control group) under different treatments of consumption and non-consumption of nitrogen, Rohitashav et al. (1993) found that the yield of dry matter of forage was higher due to inoculation benefits. Hernandez et al. (1995) also reported that inoculating seeds with nitrogen-fixing bacteria enhanced the fresh weight of aerial parts of plant and the number of leaves per plant.

### Content of Essential Oil (EO)

**Figure 1:**
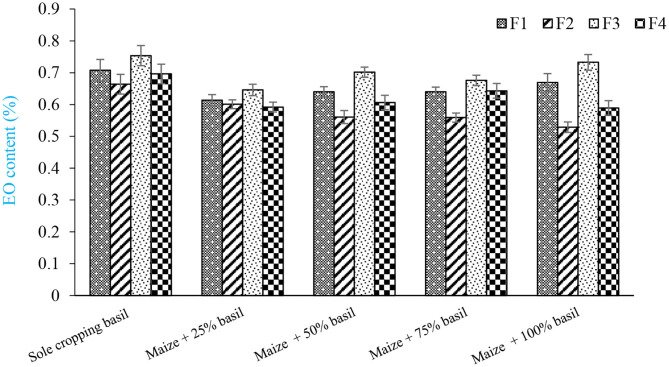
Mean comparisons of interaction of cropping patterns and N_2_ fixing bacteria on content of basil essential oil. F1, control; F2, 100% chemical fertilizer (N); F3, *Azospirillum brasilense* and *Azotobacter chroococcum*; F4, integration of *A. brasilense* and *A. chroococcum* + 50% nitrogen chemical fertilizer.

Intercropping forage maize + 100% basil with chemical nitrogen fertilizer resulted in the lowest content of EO (0.53%) while the highest (0.75%) was obtained by sole cropping of sweet basil with N_2_ fixing bacteria of *A. brasilense* and *A. chroococcum* (Figure 1). Under sole cropping pattern, competition between sweet basil plants was reduced because the space needed for growth was increased. Thus, sole cropping pattern, as compared to intercropping pattern, adequately supplied light and soil resources to increase EO content in plants. Amaki et al. (2011) stated that amount of EO of sweet basil was affected by intensity of light, as EO was enhanced by excessive light. Figueiredo et al. (2008) stated that intense sunlight directly resulting in an increase in the EO of *O. basilicum*.

In a study by Nobahar and Pazoki (2010) on sweet basil, increasing the number of plants per area significantly reduced the content of EO. A reduction in plant density resulted in an increase in percentage of EO due to reduction in the level of plants' competition. However, in our study, there was not a significant difference in EO content between intercropping patterns (forage maize + 100% basil and forage maize + 50% basil) nourished by *A. brasilense* and *A. chroococcum* and sole cropping pattern of sweet basil nourished by *A. brasilense* and *A. chroococcum*. This showed that, in addition to producing more forage maize per area which is considered as a merit of cropping medicinal forage, the quality was not negatively affected by intercropping with maize when fertilized with N_2_ fixing bacteria.

According to the results of this research, it seems that there is an inverse relationship between EO content of sweet basil and using nitrogen chemical fertilizer. The superiority of controls over other treatments, i.e., nitrogen chemical fertilizer and an integration of N_2_ fixing bacteria + 50% nitrogen chemical fertilizer with high biological yield, is attributed to an increase in secondary metabolite under environmental stress and nutritional deficiency conditions. Nitrogen chemical fertilizer and an integration of N_2_ fixing bacteria + 50% nitrogen chemical fertilizer adequately paved the way for plants to grow adequately through supplying nutritional resources. Khalediyan et al. (2020) stated that EO yield of *Ocimum basilicum and Satureja hortensis* was significantly increased due to N_2_ fixing bacteria treatments compared to control plants.

**Figure 2:**
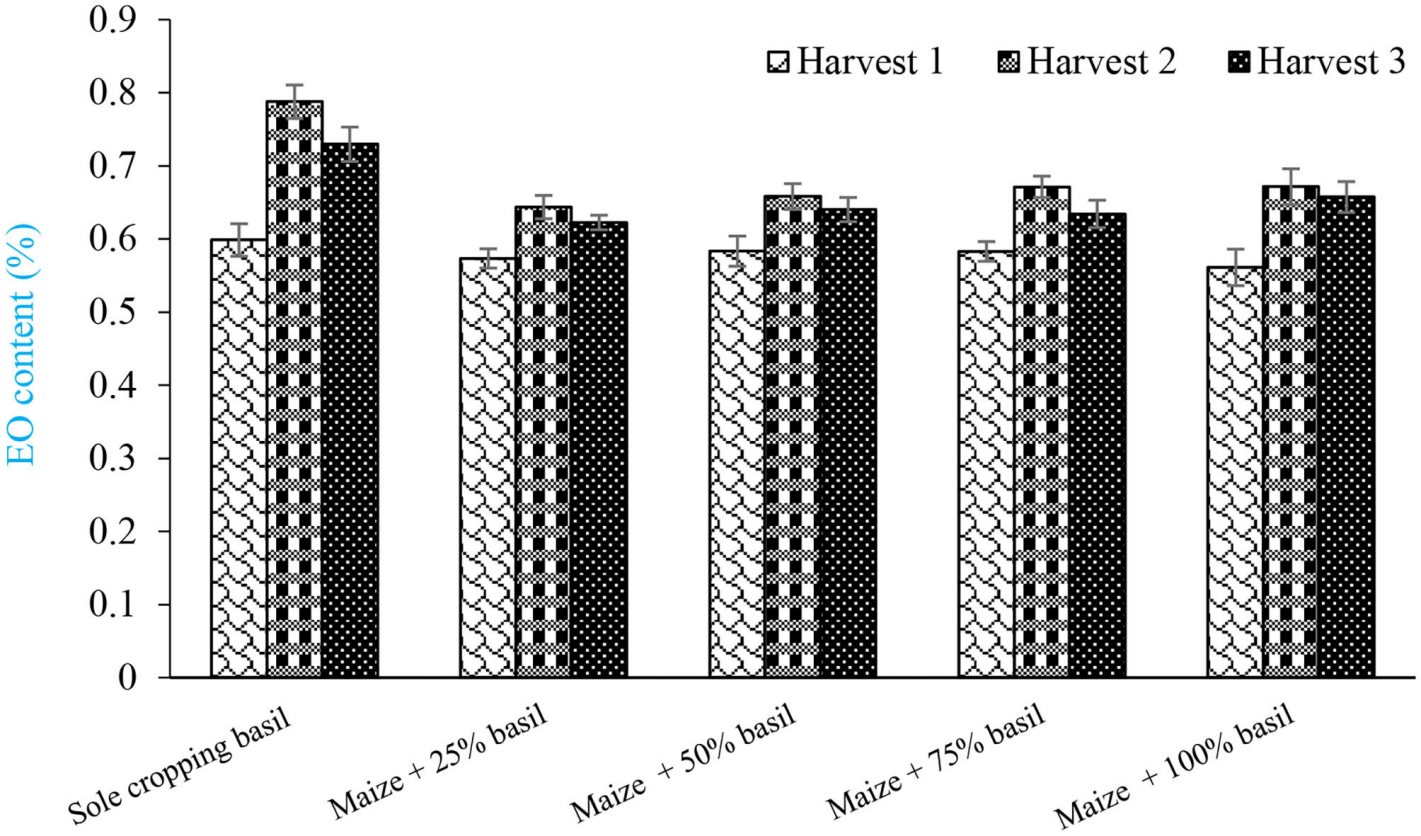
Mean comparisons of interaction of cropping patterns and different harvest on content of basil essential oil.

**Figure 3:**
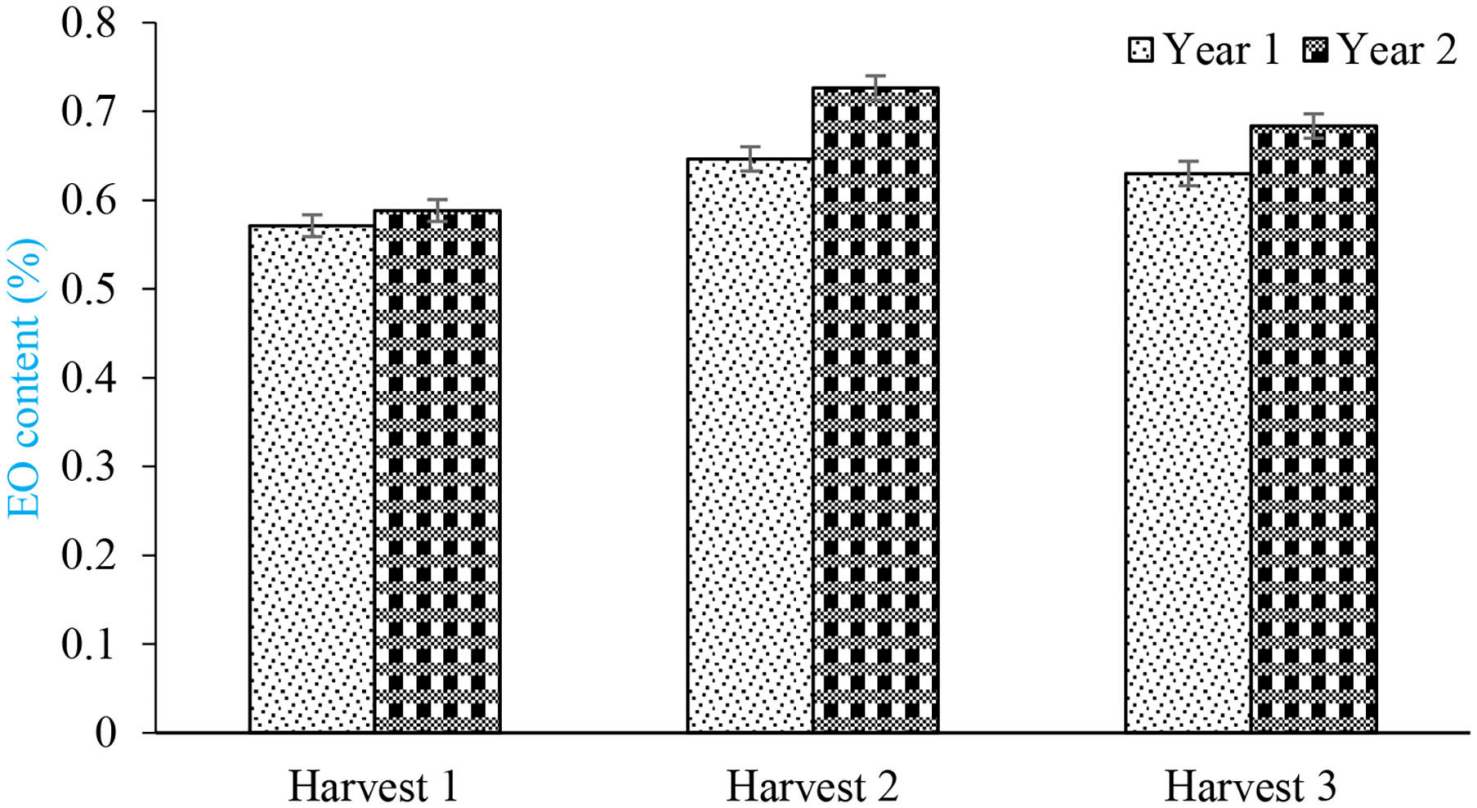
Mean comparisons of interaction of different harvest and years of experiments on content of basil essential oil.

The mean comparisons of cropping pattern × harvest showed that sole cropping of sweet basil in second harvest produced the highest content of EO (0.79%) while the lowest was recorded by intercropping forage maize + 100% basil at first harvest (0.56%) (Figure 2). Although all harvests were performed at identical growth stage, plants of second and third harvests generated more secondary metabolite due to abundant light and more photosynthesis activities. Jahan et al. (2013) stated that among all harvests, the first harvest of sweet basil produced lowest EO content. The mean comparisons of year of experiment × harvest showed that highest EO content (0.73%) was found in second harvest of second year (Figure 3). As the number of leaf and inflorescence in second year were higher than those of first year (unpublished data), and as well as more EO content of sweet basil is found in leaf and flower, it was expected that rate of EO content in second year to be higher than that of first one

### Essential Oil Yield

**Figure 4:**
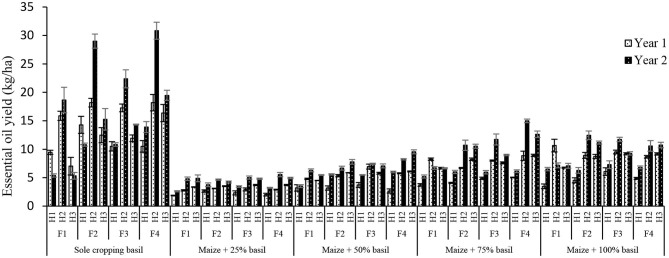
Mean comparisons of interaction of cropping patterns and N_2_ fixing bacteria in different harvest during two growing seasons on essential oil yield of basil. I1, I2, I3, I4, and I5: sole cropping of basil, the additive intercropping of maize + 25% basil, maize + 50% basil, maize + 75% basil and maize + 100% basil, respectively. F1, control; F2, 100% chemical fertilizer (N); F3, *Azospirillum brasilense* and *Azotobacter chroococcum*; F4, integration of *A. brasilense* and *A. chroococcum* + 50% nitrogen chemical fertilizer and H1, first harvest; H2, second harvest; and H3, third harvest.

The result revealed that highest yield of EO of sweet basil was achieved by sole cropping with application of N_2_ fixing bacteria + 50% nitrogen chemical fertilizer in second harvest of second year (30.8 kg ha^−1^) (Figure 4). Sole cropping yielded more EO than did other cropping practices due to lack of interspecific competition, lack of shade, and having sufficient space for extending vegetative organs in order to take advantage of available resources. These beneficial factors allowed plants to expand their height and lateral branches. In keeping with the direct relationship between light irradiation and EO production reported by Amaki et al. (2011), sole cropping provided abundant light for plants to enhance their EO content in absence of shading other taller plants (maize).

*A. brasilense* and *A. chroococcum* and control (without-fertilizer) treatments gave higher EO content compared to nitrogen chemical fertilizers and integration of *A. brasilense* and *A. chroococcum* + 50% nitrogen chemical fertilizer. This reflected the fact that EO yield of sweet basil was highly affected by biological yield and less by EO content. In other words, the contribution of EO content to increased yield of EO per area was lower than that of in biological yield of crops. This experiment confirmed the hypothesis that application of nitrogen chemical fertilizer and integrated *A. brasilense* and *A. chroococcum* + 50% nitrogen chemical fertilizer could increase yield of EO of sweet basil, mainly due to increase in biological yield. The availability of nitrogen enhances the rate of photosynthesis and enables the plant to grow rapidly and produced significant biomass, which may increase accumulation of secondary metabolites, such as EO (Sifola and Barbieri, 2006). Kumari Gour et al. (2017) stated that highest EO yield of dill was gained by using an integrated treatment of *Azospirillum* and *Azotobacter* plus 75% urea.

As the EO content and biological yield in second harvest were higher than those at the other two harvests, it was expected that the EO yield in second harvest to be higher than that of the others. Among three harvests of sweet basil, Jahan et al. (2013) demonstrated that the highest and lowest EO yield was found in second and first harvests, respectively. It was also shown that intercropping soybean with peppermint promoted the production of peppermint's EO as compared to their sole cropping and the maximum and minimum EO content were obtained in intercropping ratio of 3:2 (1.74%) and sole cropping of peppermint (1.26%), respectively. Furthermore, the first harvest produced more EO compared to the second harvest (Amani-Machiani et al., 2018). Since there is a positive correlation between EO yield and biological yield, the EO yield increased with rising density level of sweet basil plants intercropped with maize. In this respect, the maximum EO yield was attained by applying forage maize + 75% basil as well as application of *A. brasilense* and *A. chroococcum* + 50% nitrogen chemical fertilizer.

### EO Composition

**Figure 5:**
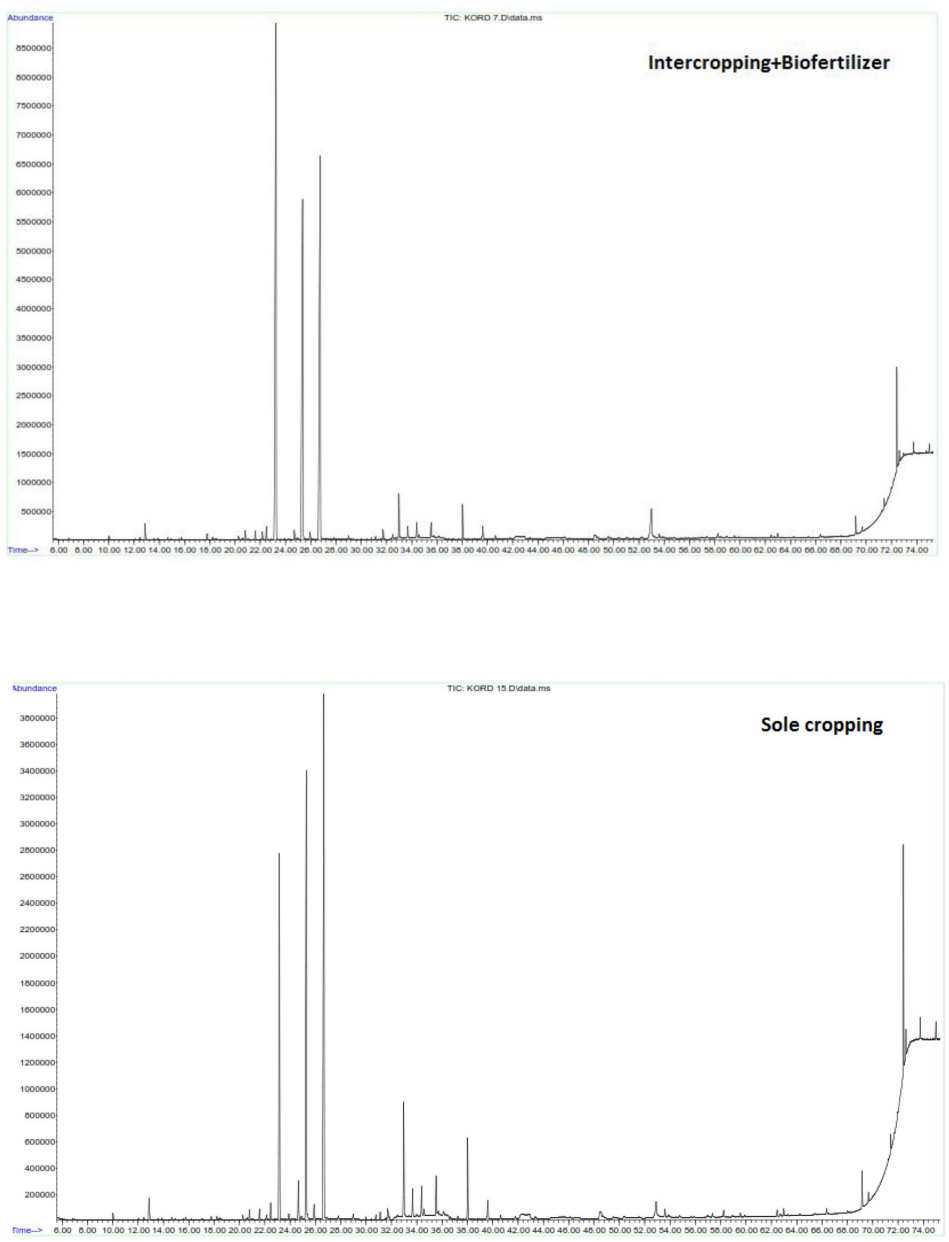
*Ocimum basilicum* L. essential oil chromatogram carried out using a GC-MS. Essential oils were obtained from non-biofertilizer with *Azospirillum brasilense* and *Azotobacter chroococcum* application and sole and intercropped plants.

In addition to quantity, the quality (in terms of type and number of constituents) of EO is also received great attentions while cultivation medicinal plants (Figure 5). The analysis of EO carried out on aerial organs of sweet basil in different treatments revealed the presence of 23–32 constituents (Tables 4, 5). As some of detected constituents such as 1,8-cineol, terpinolene, α-cubebene, eugenol, β-cubebene, β-elemen, and α-zingiberene were not observed in all treatments but found in small quantities in some treatments, they were not presented in tables. In current research, the results revealed that the types of EO components of in both years of experiments could remarkably vary depending on type of treatment. In both years, eight constituents including methyl chavicol (17.24–51.28%), Z-citral (neral) (8.33–24.3%), geranial (10.2–31.3%), (E)-caryophyllene (1.05–5.64%), α-*trans*-bergamotene (0.53–1.7%), α-humulene (0.4–1.69%), germacrene-D (0.2–1.88%), and (Z)-α-bisabolene (1.16–3.86%) were the main constituents of EO. In order to monitor the change occurred in type and amount of EO's constituents, their relative contents in different treatments were carefully assayed. The highest amount of methyl chavicol in first (51.03%) and second (51.28%) years were achieved by sole cropping of sweet basil with nitrogen chemical fertilizer (Tables 4, 5), while the lowest were obtained in first and second years (17.24 and 38.2%, respectively) by sole cropping of sweet basil without-fertilizer. In both years, the highest content of methyl chavicol was found through sole cropping of sweet basil with nitrogen chemical fertilizer followed by sole cropping of sweet basil with an integration of *A. brasilense* and *A. chroococcum* + 50% nitrogen chemical fertilizer. The availability of nitrogen in chemical fertilizer and also the lack of competition among plants of sweet basil under sole cropping seemingly increased methyl chavicol concentration compared to other treatments. Zheljazkov et al. (2008) stated that nitrogen markably changed the amount of linalool, eugenol, bornil acetate and eucalyptol of EO of sweet basil. They continued that application of more nitrogen increased methyl chavicol while it decreased linalool of EO. The highest content of Z-citral (neral) (24.3 and 16.6%), geranial (31.3 and 21.94%), and (E)-caryophyllene (5.64 and 3.27%) were observed by an intercropping of forage maize + 25% basil and without–fertilizer in first and second years of experiment, respectively. Likewise, the highest levels of α-humulene (1.69 and 1.4%), germacrene-D (1.88 and 0.65%) were found in intercropped forage maize + 100% basil without-fertilizer in first and second years of experiment, respectively (Tables 4, 5). It appears that some factors like nutrient deficiency (especially nitrogen deficiency in the control without fertilizer) and level of plants competition over light and nutrient resources under intercropping pattern could be considered as factors stimulating more production of these constituents. Main constituents of EO are affected by diverse factors: water stress, salt stress, and nutrition deficiencies resulting in change in EO constituents (Barbieri et al., 2012; Ekren et al., 2012).

**Table 4:** Essential oil constituents in basil grown with different sources of nitrogen under different patterns of cropping during 2018 growing season (expressed on relative percentage basis).

**Compounds**	**Sole cropping of basil**				**Maize** **+** **25% basil**				**Maize** **+** **50% basil**				**Maize** **+** **75% basil**				**Maize** **+** **100% basil**			
	**F1**	**F2**	**F3**	**F4**	**F1**	**F2**	**F3**	**F4**	**F1**	**F2**	**F3**	**F4**	**F1**	**F2**	**F3**	**F4**	**F1**	**F2**	**F3**	**F4**
α-Pinene	0.20	0.21	0.12	0.09	0.32	0.13	0.21	0.14	0.20	0.21	0.20	0.12	0.13	0.21	0.23	0.20	0.20	0.19	0.44	0.16
(Z)-3- Hexenyl acetate	0.05	0.10	0.16	0.10	0.03	0.06	0.13	0.06	0.06	0.13	0.09	0.14	0.11	0.12	0.08	0.11	0.12	0.13	0.09	0.12
Limonene	0.12	0.13	0.11	0.09	0.10	0.12	0.10	0.09	0.10	0.11	0.12	0.10	0.13	0.12	0.12	0.11	0.15	0.13	0.11	0.11
(E)-β- Ocimene	0.13	0.26	0.22	0.09	0.12	0.10	0.18	0.10	0.11	0.15	0.16	0.11	0.13	0.21	0.12	0.10	0.11	0.15	0.10	0.08
Fenchone	0.08	0.20	0.17	0.17	0.12	0.32	0.22	0.24	0.16	0.28	0.29	0.13	0.12	0.26	0.25	0.20	0.13	0.22	0.16	0.17
Rose furan epoxide	0.22	0.44	0.84	0.61	0.26	0.31	0.33	0.32	0.25	0.21	0.37	0.29	0.32	0.44	0.23	0.54	0.31	0.35	0.26	0.45
Methyl chavicol	**17.24**	**51.03**	**28.51**	**38.92**	**17.91**	**35.82**	**32.20**	**29.06**	**30.22**	**35.83**	**29.72**	**35.33**	**28.67**	**37.69**	**37.59**	**36.70**	**19.01**	**37.25**	**19.54**	**26.49**
Z-Geraniol (Nerol)	**0.21**	**0.68**	**0.83**	**0.57**	**1.91**	**0.70**	**1.53**	**0.44**	**0.58**	**1.27**	**0.79**	**2.32**	**2.27**	**1.93**	**0.37**	**1.19**	**2.02**	**2.60**	**1.37**	**2.51**
Z-Citral (Neral)	**21.47**	**13.92**	**22.85**	**15.77**	**24.30**	**19.07**	**21.84**	**18.46**	**21.85**	**21.84**	**22.66**	**19.42**	**22.00**	**20.11**	**20.88**	**19.70**	**19.17**	**18.46**	**20.12**	**19.60**
Geraniol	**0.85**	**0.23**	**0.34**	**0.25**	**0.76**	**0.29**	**0.81**	**0.32**	**0.85**	**0.54**	**0.40**	**0.76**	**1.02**	**0.80**	**0.42**	**0.49**	**0.82**	**1.04**	**0.50**	**0.83**
Geranial	**25.17**	**17.65**	**29.77**	**20.12**	**31.30**	**24.48**	**28.14**	**23.69**	**27.47**	**28.62**	**30.55**	**24.46**	**28.04**	**25.00**	**26.75**	**24.3**	**23.43**	**24.21**	**25.46**	**24.16**
Neryl acetate	0.17	0.38	0.73	0.24	0.26	0.15	0.19	0.18	0.21	0.17	0.15	0.34	0.36	0.23	0.18	0.55	0.39	0.35	0.22	0.51
α- Copaen	0.16	0.17	0.18	0.13	0.21	0.28	0.24	0.12	0.17	0.22	0.21	0.24	0.20	0.26	0.22	0.50	0.30	0.24	0.22	0.30
Geranyl acetate	0.09	0.09	0.22	–	–	0.09	0.40	0.06	0.06	0.10	0.49	0.10	0.15	0.10	0.30	0.20	0.16	0.08	0.10	0.18
Methyleugenol	0.11	0.46	0.18	0.25	0.24	0.31	0.19	0.20	0.32	0.20	0.16	0.18	0.13	0.25	0.20	0.42	0.17	0.19	0.17	0.24
(E)-Caryophyllene	**3.01**	**2.81**	**2.84**	**1.93**	**5.64**	**3.55**	**2.96**	**1.66**	**2.07**	**2.97**	**2.85**	**3.00**	**3.16**	**3.01**	**3.06**	**3.65**	**4.12**	**3.58**	**3.10**	**4.64**
α-trans-Bergamotene	**1.30**	**0.85**	**0.93**	**0.68**	**1.38**	**0.96**	**0.82**	**0.67**	**0.53**	**0.65**	**0.65**	**0.97**	**0.89**	**0.70**	**0.72**	**1.10**	**1.15**	**0.98**	**0.79**	**1.35**
α-Humulene	**1.55**	**1.23**	**1.35**	**0.86**	**1.50**	**1.11**	**0.92**	**0.57**	**0.70**	**0.75**	**0.96**	**0.98**	**0.97**	**1.08**	**1.02**	**1.50**	**1.69**	**1.17**	**1.03**	**1.62**
(E)- β-Farnesene	0.16	0.23	0.22	0.18	0.18	0.26	0.24	0.20	0.18	0.26	0.22	0.28	0.25	0.27	0.20	0.36	0.42	0.23	0.27	0.46
Germacrene-D	**1.50**	**0.82**	**0.70**	**0.50**	**1.87**	**1.20**	**1.29**	**0.55**	**0.61**	**0.48**	**1.03**	**1.32**	**1.28**	**1.36**	**0.68**	**1.70**	**1.88**	**1.44**	**1.00**	**1.85**
β-Bisabolene	0.10	0.13	–	0.10	0.09	0.11	0.05	0.15	0.09	0.15	0.09	0.14	0.12	0.16	0.09	0.20	0.18	0.20	0.13	0.23
(Z)-α- Bisabolene	**3.52**	**2.46**	**2.67**	**1.96**	**3.74**	**2.57**	**2.67**	**1.25**	**1.47**	**1.19**	**2.13**	**3.02**	**3.86**	**3.03**	**2.22**	**3.58**	**3.24**	**3.18**	**3.32**	**3.79**
**Caryophyllene oxide**	**0.35**	**0.84**	**1.23**	**0.58**	**1.05**	**1.02**	**0.72**	**0.85**	**0.86**	**0.73**	**0.65**	**0.74**	**0.57**	**1.11**	**1.10**	**1.10**	**0.75**	**0.68**	**0.91**	**1.04**
Humulene epoxide II	0.13	0.23	0.36	0.23	0.23	0.23	0.15	0.15	0.21	0.20	0.15	0.16	0.14	0.24	0.24	0.15	0.17	0.15	0.24	0.26

*F1, control; F2, 100% chemical fertilizer (N); F3, Azospirillum brasilense and Azotobacter cerococcid; F4, integration of A. brasilense and A. chroococcum + 50% nitrogen chemical fertilizer. Bold values show significant increases between treatments*.

**Table 5:** Essential oil constituents in basil grown with different sources of nitrogen under different patterns of cropping during 2019 growing season (expressed on relative percentage basis).

**Compounds**	**Sole cropping of basil**				**Maize** **+** **25% basil**				**Maize** **+** **50% basil**				**Maize** **+** **75% basil**				**Maize** **+** **100% basil**			
	**F1**	**F2**	**F3**	**F4**	**F1**	**F2**	**F3**	**F4**	**F1**	**F2**	**F3**	**F4**	**F1**	**F2**	**F3**	**F4**	**F1**	**F2**	**F3**	**F4**
α-Pinene	0.08	0.09	0.07	0.09	0.13	0.08	0.10	0.12	0.08	0.10	0.11	0.10	0.06	0.21	0.13	0.15	0.08	0.15	0.19	0.14
(Z)-3- Hexenyl acetate	0.08	0.13	0.11	0.12	0.06	0.09	0.09	0.08	0.08	0.15	0.09	0.15	0.10	0.17	0.08	0.14	0.10	0.16	0.10	0.12
Limonene	0.10	0.09	0.10	0.10	0.09	0.08	0.08	0.08	0.12	0.07	0.08	0.08	0.10	0.08	0.10	0.09	0.12	0.10	0.12	0.10
(E)-β- Ocimene	–	–	–	–	–	–	–	–	–	–	–	–	–	–	–	–	–	–	–	–
Fenchone	0.10	0.19	0.28	0.18	0.11	0.25	0.29	0.20	0.12	0.24	0.23	0.20	0.16	0.20	0.21	0.22	0.12	0.18	0.20	0.20
Rose furan epoxide	0.58	0.34	0.45	0.47	0.31	0.30	0.37	0.32	0.20	0.31	0.40	0.22	0.19	0.28	0.35	0.18	0.19	0.25	0.38	0.20
**Methyl chavicol**	**38.20**	**51.28**	**46.32**	**50.46**	**39.20**	**40.23**	**45.55**	**43.10**	**44.20**	**41.20**	**42.40**	**42.38**	**43.50**	**43.52**	**47.60**	**49.15**	**39.29**	**40.10**	**41.40**	**38.80**
**Z-Geraniol (Nerol)**	**0.54**	**0.35**	**0.50**	**0.60**	**0.71**	**0.40**	**1.25**	**0.70**	**0.60**	**0.90**	**1.10**	**1.19**	**0.76**	**1.22**	**1.30**	**1.10**	**0.80**	**1.50**	**1.20**	**1.60**
**Z-Citral (Neral)**	**13.48**	**8.33**	**9.79**	**15.91**	**16.60**	**13.10**	**13.59**	**16.50**	**15.90**	**15.20**	**15.60**	**9.09**	**12.70**	**14.42**	**15.41**	**8.69**	**12.50**	**9.00**	**15.29**	**8.67**
**Geraniol**	**0.28**	**0.43**	**0.28**	**0.36**	**0.23**	**0.53**	**0.53**	**0.46**	**0.30**	**0.75**	**0.60**	**0.55**	**0.38**	**0.88**	**0.50**	**0.44**	**0.25**	**0.80**	**0.48**	**0.50**
**Geranial**	**17.35**	**10.67**	**12.90**	**15.00**	**21.94**	**17.20**	**18.47**	**17.20**	**18.90**	**18.02**	**18.50**	**13.69**	**17.40**	**16.94**	**17.30**	**13.33**	**16.90**	**15.10**	**20.76**	**10.20**
Neryl acetate	0.26	0.58	0.42	0.67	0.21	0.47	0.35	0.57	0.40	0.50	0.30	0.54	0.60	0.61	0.33	0.59	0.58	0.60	0.33	0.52
α- Copaen	0.58	0.20	0.25	0.28	0.39	0.26	0.27	0.25	0.30	0.20	0.25	0.13	0.34	0.25	0.21	0.25	0.35	0.23	0.21	0.22
Geranyl acetate	0.31	0.25	0.23	0.13	0.18	0.24	0.30	0.16	0.20	0.20	0.38	0.18	0.34	0.27	0.30	0.14	0.33	0.30	0.26	0.16
Methyleugenol	0.61	2.61	1.36	0.85	1.02	1.60	1.26	0.80	1.22	0.70	1.10	1.64	0.80	0.67	1.20	1.18	0.97	0.50	0.67	1.19
(E)-Caryophyllene	**2.57**	**1.10**	**2.06**	**1.48**	**3.27**	**1.82**	**1.93**	**1.05**	**2.95**	**1.73**	**2.02**	**2.71**	**3.08**	**1.13**	**2.75**	**1.57**	**3.15**	**1.84**	**2.76**	**2.45**
α-trans-Bergamotene	**1.46**	**0.62**	**1.16**	**1.06**	**1.05**	**0.60**	**0.88**	**0.81**	**0.91**	**0.95**	**0.84**	**0.71**	**1.60**	**1.13**	**1.25**	**0.94**	**1.70**	**1.20**	**1.29**	**1.17**
α-Humulene	**1.29**	**0.43**	**1.03**	**0.96**	**0.91**	**0.40**	**0.73**	**0.70**	**0.80**	**0.44**	**1.02**	**0.40**	**1.36**	**0.48**	**1.21**	**0.69**	**1.40**	**0.57**	**1.33**	**1.21**
(E)- β-Farnesene	0.25	0.24	0.24	0.16	0.25	0.27	0.26	0.19	0.27	0.27	0.24	0.19	0.30	0.30	0.30	0.19	0.35	0.30	0.34	0.24
Germacrene-D	0.21	0.36	0.23	0.35	0.27	0.38	0.29	0.40	0.26	0.20	0.25	0.27	0.60	0.44	0.55	0.50	0.65	0.47	0.60	0.60
β-Bisabolene	0.27	0.11	0.21	0.12	0.18	0.14	0.20	0.13	0.20	0.13	0.20	0.13	0.24	0.20	0.22	0.12	0.25	0.22	0.18	0.16
(Z)-α- Bisabolene	**3.33**	**1.48**	**2.52**	**2.96**	**2.86**	**1.52**	**1.94**	**1.35**	**2.31**	**1.16**	**1.80**	**1.25**	**3.46**	**1.96**	**3.50**	**2.13**	**3.11**	**2.40**	**3.45**	**2.38**
Caryophyllene oxide	**1.20**	**1.86**	**1.63**	**2.21**	**2.09**	**2.20**	**1.31**	**2.30**	**2.00**	**2.10**	**1.20**	**2.05**	**2.07**	**2.60**	**1.35**	**1.59**	**1.80**	**2.10**	**1.20**	**1.52**
Humulene epoxide II	0.76	0.57	0.60	1.59	0.36	0.50	0.41	0.88	0.40	0.56	0.31	0.79	0.49	0.50	0.30	0.45	0.30	0.44	0.45	0.48

*F1, control; F2, 100% chemical fertilizer (N); F3, Azospirillum brasilense and Azotobacter chroococcum; F4, integration of A. brasilense and A. chroococcum + 50% nitrogen chemical fertilizer. Bold values show significant increases between treatments*.

In the first year of the experiment, the highest amount of α-*trans*-bergamotene (1.38%) was achieved by intercropping forage maize + 25% basil and without-fertilizer treatment, but in second year, highest amount (1.7%) was obtained by intercropping forage maize + 100% basil and without-fertilizer treatment. Based on Nurzyńska-Wierdak et al. (2013), different levels of nitrogen was shown to have a significant effect on the main constituents in the EO of basil in such that the highest amount α-*trans*-bergamotene in sweet basil was found by lowest level of nitrogen. At first year of experiment, the highest content of (Z)-α-bisabolene (3.86%) was obtained by intercropping forage maize + 75% basil without-fertilizer, while its highest rate in second year was found by intercropping forage maize + 75% basil and applying *A. brasilense* and *A. chroococcum* N_2_ fixing bacteria treatments (Tables 4, 5). There are some reports on effects of intercropping patterns on quality and quantity of EO. Rajeswara Rao (2002) figured out that intercropping corn mint (*Mentha arvensis* L.) with tomato under different planting dates, in addition to achieving an acceptable corn mint yield, increased the constituents' quality of corn mint EO up to different extents such as menthol (73%), menthone (9.6%), isomenthone (4%), menthyl acetate (4%). Amani-Machiani et al. (2018) showed that the highest and lowest menthol, germacrene-D and (E)-caryophyllene were gained by intercropping peppermint with soybean and sole cropping of peppermint, respectively.

Compared to first year of experiment, all treatments gained markedly higher methyl chavicol in second year of experiment. By contrast, the rate of Z-citral (neral), geranial, germacrene-D and (E)-caryophyllene were higher in the first year of experiment. Although (E)-β-ocimene was observed in all treatments in the first year, it was not observed in second year. As the relative percentage of EO constituents usually changed (even) daily and it also was affected by a great deal of factors, it is expected that compositions fluctuated during both years of experiments, however the main constituents were identical in both years. The lowest content of main constituents in EO, except methyl chavicol, was observed in a cropping pattern in which nitrogen chemical fertilizer was employed. Thus, application of *A. brasilense* and *A. chroococcum* and an integration of N_2_ fixing bacteria + 50% nitrogen chemical fertilizer is advisable to enhance the quality of EO. Plant growth-promoting bacteria (PGPB), as a growth stimulator, is able to change environmental conditions and constituents of EO with different mechanisms such as increasing nitrogen fixation and available nitrate, producing auxin and gibberellin, secreting antibiotics, and enhancing root growth and development as well as promoting water and nutrients uptake (Prasad et al., 2011; Santoro et al., 2011).

### Land Equivalent Ratio (LER)

As can be seen, in all treatments the LER values were higher than 1 (Table 6), which indicates advantage of intercropping relative to sole cropping. Meanwhile, N_2_ fixing bacteria application significantly enhanced LER in both 2018 and 2019. The maximum LER (1.56) was achieved from maize + 100% sweet basil + N_2_ fixing bacteria in 2018 and the minimum (1.17) in maize + 25% sweet basil + without fertilizer in 2019 (Table 6). The great efficiency of the intercropping systems found in the present research as supported by higher total LER is consistent with Baumann et al. (2001) findings, who attributed this phenomenon to the supplementary use of resources in plant production allowing an interspecific facilitation. Based on the findings of Hauggaard-Nielsen et al. (2003), the calculated LER demonstrated that plant growth resources were used from 27 to 31% more efficiently by intercrop than the sole crop. Khalediyan et al. (2020) indicated that LER, land utilization efficiency (LUE), relative value total (RVT) and time equivalent ratio (ATER) in intercropping were more than sole cropping.

**Table 6:** Land equivalent ratio (LER) in intercropping treatments of maize with sweet basil and N_2_ fixing bacteria (2018 and 2019).

**Treatments**	**2018**	**2019**
Maize + 25% sweet basil-without fertilizer	1.28	1.17
Maize + 25% sweet basil+ chemical fertilizer	1.22	1.29
Maize + 25% sweet basil + *A. brasilense* and *A. chroococcum*	1.31	1.23
Maize + 25% sweet basil + *A. brasilense* and *A. chroococcum* + 50% chemical fertilizer	1.25	1.16
Maize + 50% sweet basil-without fertilizer	1.38	1.38
Maize + 50% sweet basil + chemical fertilizer	1.34	1.33
Maize + 50% sweet basil + *A. brasilense* and *A. chroococcum*	1.46	1.41
Maize + 50% sweet basil + *A. brasilense* and *A. chroococcum* + 50% chemical fertilizer	1.38	1.33
Maize + 75% sweet basil and control	1.46	1.52
Maize + 75% sweet basil + chemical fertilizer	1.45	1.43
Maize + 75% sweet basil + *A. brasilense* and *A. chroococcum*	1.55	1.54
Maize + 75% sweet basil + *A. brasilense* and *A. chroococcum* + 50% chemical fertilizer	1.49	1.46
Maize + 100% sweet basil-without fertilizer	1.54	1.52
Maize + 100% sweet basil +chemical fertilizer	1.51	1.47
Maize + 100% sweet basil + *A. brasilense* and *A. chroococcum*	1.56	1.56
Maize + 100% sweet basil + *A. brasilense* and *A. chroococcum* + 50% chemical fertilizer	1.52	1.48

## Conclusion

The results of this research showed that the biological yield and EO yield of sweet basil intercropped with maize was enhanced as plant density was increased, such that among different intercropping patterns the maximum biological yield and EO yield were found in treatments with higher sweet basil plants density intercropped with maize. Compared to nitrogen chemical fertilizer, an integration of *A. brasilense* and *A. chroococcum* + 50% nitrogen chemical fertilizer showed an acceptable potential in terms of biological yield; and this treatment did not show a remarkable difference with nitrogen chemical fertilizer. The highest yield of EO was attained by applying *A. brasilense* and *A. chroococcum* + 50% nitrogen chemical fertilizer, although N_2_ fixing bacteria and no-fertilizer treatments gave higher EO content than did nitrogen chemical fertilizer and *A. brasilense* and *A. chroococcum* + 50% nitrogen chemical fertilizer treatments, but nitrogen chemical fertilizer and an integration of *A. brasilense* and *A. chroococcum* + 50% chemical fertilizer treatments gave higher EO yield. This can be justified as EO yield was mainly affected by biological yield rather than EO content. As the lowest concentrations of main constituents of EO, except methyl chavicol, were obtained by a cropping pattern in which nitrogen chemical fertilizer was used, it is recommended to use the cropping pattern in which *A. brasilense* and *A. chroococcum* or an integration of *A. brasilense* and *A. chroococcum* + 50% nitrogen chemical fertilizer to be utilized. These treatments increased the quality and quantity of generated EO. Also, the highest contents of main EO constituents, except methyl chavicol, were obtained by intercropping; and this indicated an increase in content of most main EO constituents under intercropping sweet basil with forage maize, as compared to its sole cropping.

## Data Availability Statement

The original contributions presented in the study are included in the article/supplementary material, further inquiries can be directed to the corresponding author.

## Author Contributions

SK, SS, JK, WW, and DS designed the study. SK, SS, and JK conducted the experiment and analyzed the data with support from WW and DS wrote the paper with contributions from all co-authors. All authors contributed to the article and approved the submitted version.

## Conflict of Interest

The authors declare that the research was conducted in the absence of any commercial or financial relationships that could be construed as a potential conflict of interest.
